# Ribosomal DNA arrays are the most H-DNA rich element in the human genome

**DOI:** 10.1093/nargab/lqaf012

**Published:** 2025-03-04

**Authors:** Nikol Chantzi, Candace S Y Chan, Michail Patsakis, Akshatha Nayak, Austin Montgomery, Ioannis Mouratidis, Ilias Georgakopoulos-Soares

**Affiliations:** Institute for Personalized Medicine, Department of Biochemistry and Molecular Biology, The Pennsylvania State University College of Medicine, 500 University Drive, C5716 Hershey, PA 17033, USA; Institute for Personalized Medicine, Department of Biochemistry and Molecular Biology, The Pennsylvania State University College of Medicine, 500 University Drive, C5716 Hershey, PA 17033, USA; Institute for Personalized Medicine, Department of Biochemistry and Molecular Biology, The Pennsylvania State University College of Medicine, 500 University Drive, C5716 Hershey, PA 17033, USA; Institute for Personalized Medicine, Department of Biochemistry and Molecular Biology, The Pennsylvania State University College of Medicine, 500 University Drive, C5716 Hershey, PA 17033, USA; Institute for Personalized Medicine, Department of Biochemistry and Molecular Biology, The Pennsylvania State University College of Medicine, 500 University Drive, C5716 Hershey, PA 17033, USA; Institute for Personalized Medicine, Department of Biochemistry and Molecular Biology, The Pennsylvania State University College of Medicine, 500 University Drive, C5716 Hershey, PA 17033, USA; Institute for Personalized Medicine, Department of Biochemistry and Molecular Biology, The Pennsylvania State University College of Medicine, 500 University Drive, C5716 Hershey, PA 17033, USA

## Abstract

Repetitive DNA sequences can form noncanonical structures such as H-DNA. The new telomere-to-telomere genome assembly for the human genome has eliminated gaps, enabling examination of highly repetitive regions including centromeric and pericentromeric repeats and ribosomal DNA arrays. We find that H-DNA appears once every 25 000 base pairs in the human genome. Its distribution is highly inhomogeneous with H-DNA motif hotspots being detectable in acrocentric chromosomes. Ribosomal DNA arrays are the genomic element with a 40.94-fold H-DNA enrichment. Across acrocentric chromosomes, we report that 54.82% of H-DNA motifs found in these chromosomes are in rDNA array loci. We discover that binding sites for the PRDM9-B allele, a variant of the PRDM9 protein, are enriched for H-DNA motifs. We further investigate these findings through an analysis of PRDM-9 ChIP-seq data across various PRDM-9 alleles, observing an enrichment of H-DNA motifs in the binding sites of A-like alleles (including A, B, and N alleles), but not C-like alleles (including C and L4 alleles). The enrichment of H-DNA motifs at ribosomal DNA arrays is consistent in nonhuman great ape genomes. We conclude that ribosomal DNA arrays are the most enriched genomic loci for H-DNA sequences in human and other great ape genomes.

## Introduction

The right-handed double-helix structure of DNA, known as B-DNA, was discovered in 1953. Since then, >20 noncanonical DNA secondary structures have been reported, including G-quadruplexes, hairpins, cruciforms, and triplexes [1]. Sequences that predispose the DNA to noncanonical conformations are known as non-B DNA motifs. Non-B DNA motifs are highly abundant in the human genome and have been associated with a number of biological functions [2–8].

Mirror repeats consist of two consecutive copies of the same sequence, with one copy being in reverse orientation, separated by an intervening spacer sequence, which lacks symmetry. AG/TC-rich mirror repeats have been shown to fold in intramolecular triple-stranded DNA, also known as H-DNA, in which a strand of DNA with mirror symmetry folds back to itself [9–11]. Mirror repeat sequences capable of adopting H-DNA conformations are frequently found in mammalian genomes. In the human genome, these sequences have been estimated to occur approximately once every 50 000 base pairs (bps) [12]. H-DNA has been characterized for its functional roles in different biological processes including in gene regulation and in DNA replication [13–19]. The formation of H-DNA at *MYC* promoter has been shown to interfere with transcription [13, 20]. Studies have also shown that H-DNA is a mutational hotspot for human diseases including cancer [2, 21–23]. For instance, the expansion of GAA·TTC repeats that can form triplex structures have been linked to the etiology of Friedreich’s ataxia [24, 25].

The genes that encode 45S ribosomal DNA (rDNA) are arranged in long tandem repeats located on the short arms of the acrocentric chromosomes and transcribed by RNA Polymerase I (RNAPI) [26, 27]. Previous work has indicated the presence of H-DNA motifs in human rDNA arrays [28]. Studies have also located triplex targeting sites at rDNA loci, which can modulate gene expression [29]. Other studies have found long noncoding RNAs that emerge at rDNA loci, and which can form intermolecular triplex structures at rDNA sites to control the epigenetic state of rDNA genes [30, 31]. Nevertheless, the systematic study of rDNA arrays and the nearby intergenic loci has been hindered by the lack of sequencing technologies that can resolve these most repetitive genomic sequences of the human genome. In the Genome Reference Consortium’s Human Build 38 the incomplete genomic regions amounted to ∼8% [32], underscoring gaps in our understanding of the genome.

The recent completion of the human genome through the telomere-to-telomere (T2T) consortium [32] enables the examination of the distribution and frequency of repeat elements in regions of the genome that were only partially annotated previously, including centromeres, telomeres, and rDNA arrays. Among these regions, rDNA arrays were previously not resolved due to their highly repetitive nature. A typical diploid human genome contains an average of 315 rDNA copies, with a standard deviation of 104 copies [33], while the CHM13 reference genome identified ∼400 copies [32]. In the T2T consortium, rDNA arrays were described as the most complex region of the CHM13 graph and their determination was a major milestone [32]. PRDM9 is a protein involved in genetic recombination and the positioning of recombination hotspots during meiosis [34]. PRDM9 is highly variable in the human population [35, 36] and in mice [37, 38]. In a study, Guarracino *et al.* showed that binding sites specific to the PRDM9-B allele, one variant of the PRDM9 protein, can be identified at rDNA and mediate frequent double-stranded breaks during meiosis [39]. Other recent efforts to assemble T2T organismal genomes are also ongoing. For instance, recent studies have completed the generation of T2T assemblies for multiple nonhuman primate species through which we can gain insights into the diversity, evolution, and plasticity of different repeats in the primate lineage [40, 41].

Here, we examined the distribution of H-DNA in human chromosomes using the recent T2T CHM13 gap-less assembly. We report that rDNA arrays have the highest genome-wide density of H-DNA motifs in the human genome (40.94-fold enrichment over background rates) and are orders of magnitude more H-DNA rich than any other genomic compartment. Across the rDNA array copies, the H-DNA motifs are preferentially positioned in specific sites, primarily at the intergenic spacer regions. We show that this phenomenon is consistent in other great ape genomes. We found that binding sites for PRDM9B, a variant of PRDM9, a protein highly represented at rDNA loci and crucial for regulating double-strand break formation and defining meiotic recombination hotspots in humans and most mammals, are enriched with H-DNA motifs. We conclude that ribosomal DNA arrays contain the highest concentration of H-DNA motifs in the human genome.

## Materials and methods

### Data retrieval

We downloaded the reference human genome assembly T2T-CHM13v2.0. Associated files including gene annotation and comprehensive centromere/satellite repeat annotation files were downloaded from https://github.com/marbl/CHM13. The gene annotation GFF file was downloaded from https://ftp.ncbi.nlm.nih.gov/genomes/all/GCF/009/914/755/GCF_009914755.1_T2T-CHM13v2.0/GCF_009914755.1_T2T-CHM13v2.0_genomic.gff.gz, and a more comprehensive centromere/satellite repeat annotation file was derived from https://s3-us-west-2.amazonaws.com/human-pangenomics/T2T/CHM13/assemblies/annotation/chm13v2.0_censat_v2.0.bed.

We also downloaded T2T genomes for the following primates [40, 41]: *Gorilla gorilla, Pan paniscus*, *Pan troglodytes*, *Pongo abelii*, *Pongo pygmaeus*, and *Symphalangus syndactylus* from https://github.com/marbl/Primates. Associated files including genome annotations were downloaded from https://github.com/marbl/t2t-browser, from which we also derived annotations for centromere/satellite repeats and gene annotations.

PRDM9B-binding sites throughout the human genome were derived from https://zenodo.org/records/7692555/files/SupplementaryFile7.chm13v2.PRDM9.tsv?download=1 as estimated in Guarracino *et al.* [39]. The RepeatMasker annotation of CHM13v2 was derived from https://s3-us-west-2.amazonaws.com/human-pangenomics/T2T/CHM13/assemblies/annotation/chm13v2.0_RepeatMasker_4.1.2p1.2022Apr14.out from which we derived Alu and SVA repeats.

The gene coordinates in T2T-CHM13v2.0 were extracted from the GFF RefSeq annotations which can be downloaded using the following ftp link: https://ftp.ncbi.nlm.nih.gov/genomes/all/GCF/009/914/755/GCF_009914755.1_T2T-CHM13v2.0//GCF_009914755.1_T2T-CHM13v2.0_genomic.gff.gz

### Identification of H-DNA motifs

A mirror repeat was defined as a sequence repeated with a center of symmetry on the same strand with arm sizes of at least 10 bp and spacer lengths of <8 bp. A subset of mirror repeats, known as H-DNA, is predisposed to forming triple helical structures through Hoogsteen bonds. H-DNA sequences were defined with a high AG/CT content exceeding 90%, arm lengths of at least 10 bp, spacer sizes of <8 bp, and excluding sequences with ≥0.8 total AT content, in accordance with literature [12, 42]. Mirror repeats were identified with the tool non-B-gfa [42] and H-DNA motifs were filtered based on the selected parameters. Furthermore, consensus sequences containing N or other characters not originating from the nucleotide alphabet {“A,” “G,” “C,” “T”} were filtered out from the extracted H-DNA dataset. The frequency of mirror repeats and H-DNA motifs with different spacers and arms was calculated.

H-DNA motif density was calculated as the number of H-DNA motif bps over the number of base pairs examined. The H-DNA motif density across genomic subcompartments was assessed by calculating the ratio of the length of H-DNA motif overlaps to the total length of each subcompartment. Subcompartment coordinates were derived from the corresponding GFF files, and any overlapping annotations within a subcompartment were consolidated.

Genes harboring H-DNA motifs with arm lengths ≥60 bps were used to perform GO term analysis with ShinyGO [43]. H-DNA motifs at endogenous repeat elements were identified with RepeatMasker. G-quadruplexes were detected using G4Hunter [44] as described in [44, 45]. Overlap between H-DNA, G-quadruplex, and endogenous repeat elements was performed with BEDTools [46].

### H-DNA density in nonhuman primate species

For each primate we downloaded the diploid file from genome ark. The H-DNA density in primates was calculated genome-wide as the total number of nonoverlapping H-DNA base pairs to the genome size. For each primate, we downloaded the respective big bed files from genome ark containing centromeric, pericentromeric compartments, and genic regions. By merging overlapping components from each distinct region, we calculated the average H-DNA density, while denoting the genome average with a dashed line. Thus, the enrichment always refers to the average H-DNA density over the genome-wide estimated density. In these fasta files, a part of the rDNA regions appears as a distinct sequence ID, which we excluded from the analysis.

### TSS/TES density plots

For each protein coding gene present in the GFF T2T-CHM13v2 file, we expanded each transcription start and transcription end site (TES) around a 3-kB window. For each site, using bedtools intersect we calculated the H-DNA motifs that overlapped within the expanded window and, subsequently, using a Python script, we calculated the total number of H-DNA bp counts at a distance relative to the transcription start sites (TSS) and TES, respectively. Finally, the resulting arrays were divided over the window average to estimate the local enrichment relative to the transcription start and end sites. The exact procedure was used to generate the density plots for the nuclear ribosomal RNA (rRNA) genes located within rDNA arrays, with the sole exception that we used the 18S and 28S genes to estimate the density.

### Generation of genomic bins

For each chromosome for the human T2T genome, we generated *N* = 2000 genomic bins of equal length. Each H-DNA start and end coordinate was assigned at a particular bin from 1 to 2000, according to the following formula:


\begin{equation*}bin\left( x \right) = 1 + {\rm floor}\left( {\frac{{2000x}}{{{\rm chromosome}\quad {\rm size}}}} \right)\end{equation*}

where *x* is the start or end coordinate of the H-DNA motif. Subsequently, for each bin the total number of H-DNA occurrences was estimated by summing the total distinct start or end coordinates within a particular bin. In cases where the start and the end of the H-DNA were assigned a different bin, the H-DNA was counted in all the intermediate bins. We calculated the number of bps covered by H-DNA motifs, for each H-DNA spacer length.

The logoplots were constructed by keeping only the arm sequences of the H-DNA motifs at the vicinity of the TSS (±3000 bp) and at the vicinity of the TES (±3000 bp) and estimating the most frequent arm sequences across all instances of H-DNA motifs.

We also examined the positioning of H-DNA motifs by partitioning the broader rDNA array in 3500 equally sized bins. The H-DNA motifs table was filtered in order to contain solely H-DNA motifs belonging to the corresponding rDNA array on each chromosome. The highlighted areas correspond to rRNA genic compartments derived from the respective GFF NCBI annotations. In particular, depending on the position of the genic rRNA compartment in the adjacent rDNA array, different colors were used to indicate 18S, 5.8S, and 28S, respectively. Furthermore, we calculated the total number of H-DNA motifs occurring in each bin in the rDNA array and the final enrichment was derived by dividing the total number of observed H-DNA across the spanning rDNA array.

### Estimation of H-DNA density relative to PRDM9B-binding sites

To investigate the relationship between H-DNA motifs and PRDM9B-binding sites, we generated local windows around the center of the PRDM9B-binding sites and measured the distribution of H-DNA bps across the window. Prior to density estimation, the PRDM9 overlapping motifs were filtered, keeping those with a False Discovery Rate (FDR) (*q*-value) score less strictly than 0.005, and, additionally, they were merged into a superset interval using *bedtools merge*. The enrichment was calculated as the number of occurrences at a position over the mean number of occurrences across the window.

We derived the raw FASTQ read files for PRDM9B and the associated controls for the HEK293T cell line from Altemose *et al.* (BioProjectPRJNA388401) [47]. Our experimental data originated from the runs SRR5627138, SRR5627139, and SRR5627140. All raw FASTQ read files were downloaded using sra-toolkit using prefetch and fasterq-dump commands. Subsequently, we ran the nf-core ChIP-seq pipeline using two different sets of parameters. For the first peak calling analysis, we used the default bwa aligner, broad peak detection, and blacklisted low complexity regions and repetitive regions for CHM13v2. However, since we were also interested in investigating the PRMD9B-binding sites in rDNA satellites, we performed another run of the nf-core ChIP-seq pipeline using parameters optimized for repetitive sequences. This time, due to inherent high repetitiveness of rDNA satellite regions, we utilized bowtie2 aligner along with the appropriate flags to keep duplicates and reads mapping to multiple regions. For both runs, we used 60 GB memory and 32 CPUs. We provide the commands below:

nextflow run nf-core/chipseq –input samplesheet_withReps.csv –outdir $OUTDIR –gtf $PATH/GCF_009914755.1_T2T-CHM13v2.0_genomic_replaced.gtf.gz –fasta $PATH/chm13v2.0.fa.gz –blacklist $BLACKLIST -profile singularity –saveReference –read_length 50 –skip_qc –skip_preseq –max_cpus 32 –max_memory 60.GB nextflow run nf-core/chipseq –input samplesheet_withReps.csv –outdir $OUTDIR –gtf $PATH/GCF_009914755.1_T2T-CHM13v2.0_genomic_replaced.gtf.gz –fasta $PATH/chm13v2.0.fa.gz -profile singularity –saveReference –read_length 50 –aligner bowtie2 –keep_multi_map –keep_dups –blacklist $BLACKLIST –skip_qc –skip_preseq –max_cpus 32 –max_memory 60.GB

After merging overlapping broad peaks from the individual replicates, we intersected the reported broad peaks from both replicates, keeping the regions with at least 20% reciprocal overlap. The PRDM9B enrichment analysis was performed as described for the PRDM9B motif, with position zero corresponding to the center of the overlapping broad peaks. In particular, for the PRMD9B enrichment analysis in rDNA, since the two replicates had multiple nonoverlapping regions, we filtered those that exhibited 75% reciprocal overlap. Statistical significance of the association between PRDM9B binding and H-DNA motif presence at rDNA loci was estimated using Fisher’s exact test.

For the called peaks that originated from both Chip-Seq pipelines, we used *bedtools intersect* utility to determine the number of the total H-DNA motifs that overlap with the called peaks.

### Investigation of H-DNA motif enrichment for different PRDM9 alleles

We analyzed ChipSeq data reported by Alleva *et al.* [35]. These data were collected in eight individuals with various PRDM9 genotypes, either homozygous (A/A), or heterozygous (A/B, A/N, A/C, or C/L4). For each individual, we used liftover to map the PRMD9 called peaks in hg19 to the T2T-CHM13v2.0 genome. Subsequently, we took the middle of each ChIP-seq peak and expanded into a 3 kB window around the center. After merging the overlapping H-DNA motifs, for each expanded interval we calculated the overlaps using *bedtools intersect* for the extracted H-DNAs. Finally, for each overlap, we calculated the occurrences of H-DNA motifs at each position relative to the PRMD9 mid from −3 kB to +3 kB. The resulting array (−3 kB and 3 kB) was divided by the window average occurrences to evaluate the H-DNA enrichment at each position. The 95% confidence intervals were calculated following the procedure–as described above– using bootstrap *N* = 1000 with replacement. At each position relative to the PRMD9 mid, the lower confidence interval represents the 0.25 quantile, whereas the top end represents the 0.975 quantile of the enrichment across the bootstrapped samples. The barplot in Fig. 5C represents the maximum enrichment of H-DNA density across the 6 kB interval relative to the mid of the PRMD9 peak. If the maximum H-DNA enrichment occurred within 250 bp of the PRMD9 mid, then we colored it in green, whereas in gray appear maximum enrichments that occurred outside of the 250 bp window.

### Examination of overlap between short tandem repeats and H-DNA motifs

Short tandem repeats (STRs) were detected using RPTRF, without mismatches in the repeats [48], with parameters maximum motif size, *M* = 50 000, and minimum length, *t* = 1 as described in [49].

## Results

We analyzed the occurrence of mirror repeats, sequences that are repeated with a center of symmetry on the same strand, throughout the T2T reference human genome. We find a total of 1 256 992 mirror repeats, characterized by arm lengths of at least ten bps and spacer lengths <8 bp. We selected the subset of mirror repeats with high AG/CT content and small spacer lengths, which can form Hoogsteen bonds and fold in H-DNA structures [50]. Specifically, we chose the mirror repeats that were AG-rich/CT-rich (≥90% AG/CT content), and filtered out sequences with high AT content (≥80%), in accordance with literature [12, 42]. Using these criteria, we report a total of 235 970 H-DNA motifs, genome-wide. The subset of H-DNA loci that are nonoverlapping are 123 604 mutually exclusive genomic regions across the human genome. These constitute 39.65 H-DNA motifs per megabase (mB) or 1588.88 H-DNA bps per mB. Previous studies had estimated the presence of an H-DNA motif every 50 000 bps [12]; however, here we report that based on the complete human reference genome that has resolved sequencing gaps and assembled repetitive sequences, there are ∼1.98 H-DNA motifs per 50 000 bps or 1 H-DNA motif roughly every 25 000 bps.

**Figure 1.**
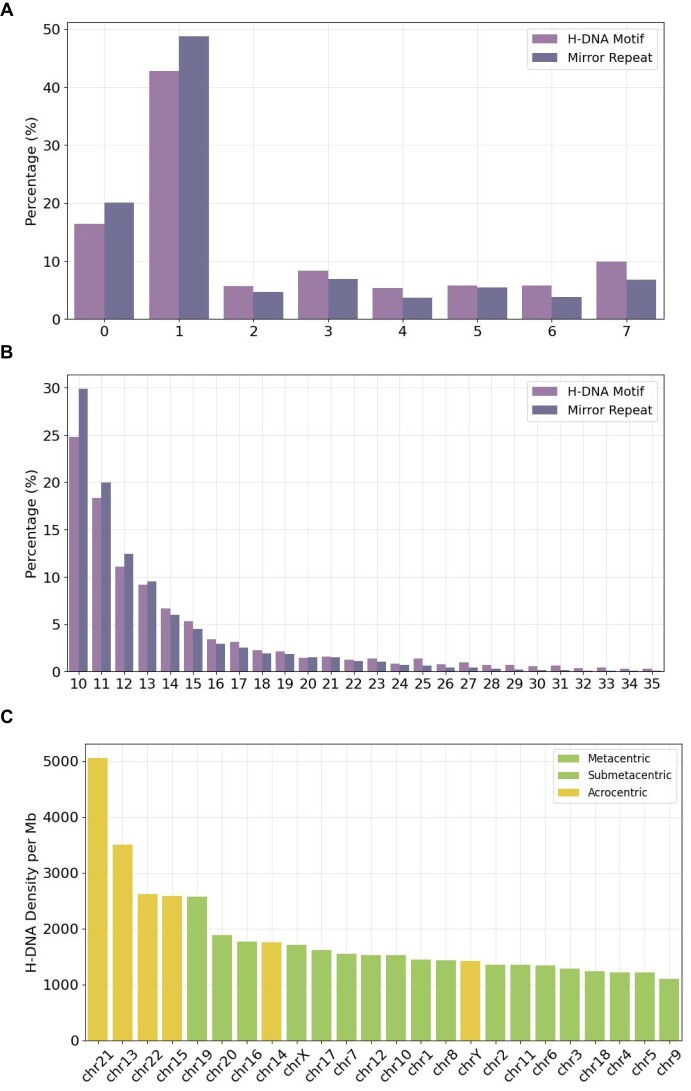
Distribution of mirror repeats, H-DNA motifs in the human genome, across chromosomes. (**A**) Distribution density of mirror repeats, H-DNA motifs as a function of spacer length. (**B**) Distribution density of mirror repeats, H-DNA motifs as a function of arm length. (**C**) Distribution density of H-DNA motif occurrences across human chromosomes. Acrocentric chromosomes with rDNA array loci are colored differently.

We were interested to examine the frequency of mirror repeats and H-DNA as a function of spacer and arm lengths. Both mirror repeats and H-DNA motifs show a preference for short spacer lengths. When comparing the distributions of H-DNA motifs to mirror repeats, we find that H-DNA motifs prefer longer spacer lengths (Fig. 1A). Additionally, we subdivided mirror repeats and H-DNA as a function of arm length (Fig. 1B). We observe that as expected, the number of mirror repeats and H-DNA motifs declines precipitously with arm length but do not find statistically significant differences between the density distributions of the arm lengths in mirror repeats and H-DNA motifs (Kolmogorov–Smirnov test, *P*-value > 0.05). We also find that the longest mirror repeat was an H-DNA with arm length of 797 bp and spacer length of 1 bp.

Interestingly, we find several H-DNA motifs that span hundreds of bps are found within genes (Supplementary Table S1). Notably, the longest H-DNA sequence of 797 bp lies entirely within the *NT5C1A* (5′-Nucleotidase, Cytosolic IA), which is associated with Myositis [51]. Furthermore, an H-DNA of 263 bps lies entirely within the gene *MID2*, which is associated with Intellectual Disability [52]. Furthermore, using a cutoff of 60 bp arm length, we find 99 genes that harbor large H-DNA sequences (Supplementary Data). We performed a GO term analysis of those genes and for cellular component terms, we found terms of the central nervous system which are consistent with previous research (Supplementary Fig. S1) [53]. Additionally, the GO biological process did not yield any significant enrichment, whereas the GO molecular function revealed a 7.4-fold enrichment in the GTPase activator activity pathway. We conclude that H-DNA motifs are more abundant in the human genome than previously estimated, with a small subset having unusually large arm lengths.

Next, we were interested in investigating potential differences in the distribution of H-DNA between human chromosomes. We observe marked differences in the density of H-DNA motifs between human chromosomes (Fig. 1C). We find that four out of the five acrocentric chromosomes showed the highest H-DNA genomic densities, with highest densities among them being observed in chromosomes 21 and 13. This led us to further investigate the reason for the increased frequency of H-DNA sequences in acrocentric chromosomes.

### H-DNA motif frequency is biased between human chromosomes and exhibits hotspots

We investigated if the distribution of H-DNA motifs is uniform throughout the human genome or if it exhibits hotspots and coldspots, loci with either an enrichment or a depletion of H-DNA motifs, respectively. We split each human chromosome in 2000 consecutive, nonoverlapping genomic bins of equal length and examined the frequency of H-DNA motifs in each of the bins.

**Figure 2.**
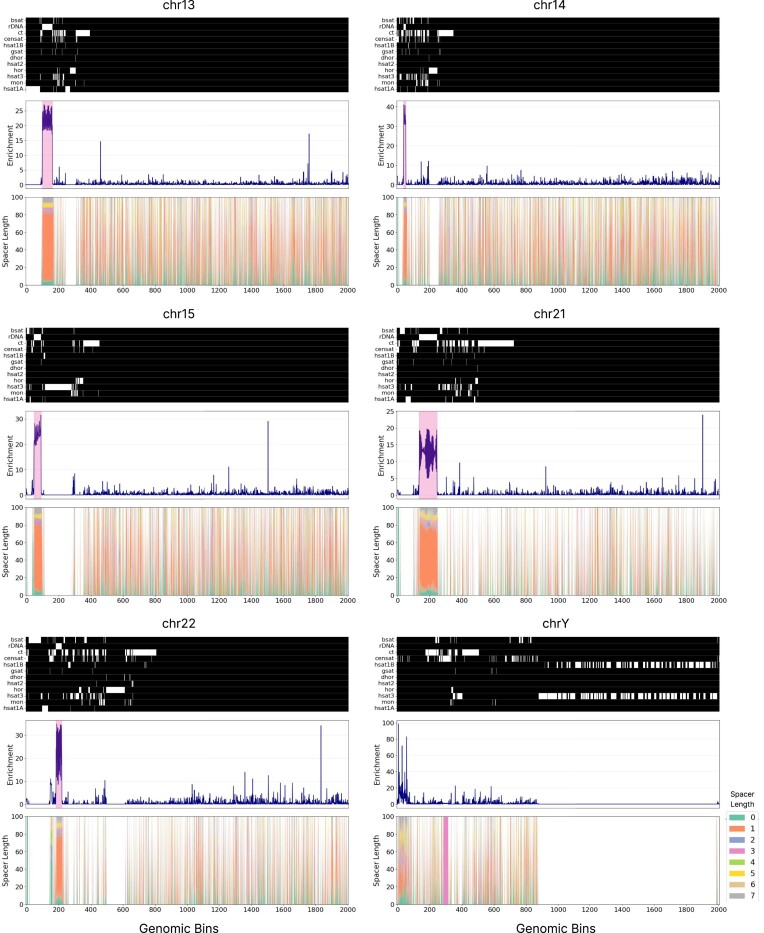
Characterization of H-DNA motifs across chromosomes in the T2T reference human genome. Schematics show the distribution of H-DNA motifs across different human chromosomes. The heatmap shows the different types of pericentromeric, centromeric repeats as well as rDNA arrays, with white representing presence in that genomic region. Line plots show the H-DNA motif enrichment at each genomic bin for a chromosome. Highlighted are the rDNA array loci. Repeats include inactive αSat HOR (hor), divergent αSat HOR (dhor), monomeric αSat (mon), classical human satellite 1A (hsat1A), classical human satellite 1B (hsat1B), classical human satellite 2 (hsat2), classical human satellite 3 (hsat3), β-satellite (bsat), γ-satellite (gsat), other centromeric satellites (censat), and centromeric transition regions (ct).

Interestingly, we observe a highly inhomogeneous distribution of H-DNA motifs across the human chromosomes and throughout the genomic bins at individual chromosomes. We find that acrocentric chromosomes, including chromosomes 13, 14, 15, 21, and 22 exhibit localized enrichment peaks for H-DNA sequences, in specific consecutive bins. The highest H-DNA motif enrichments were observed for chromosomes 14 and 22, both showing sharp peaks exceeding 35-fold above background rates. In contrast, chromosomes 15, 21, and 22 had enrichments ∼15- to 25-fold fold above background rates, but these hotspots covered larger genomic regions of consecutive genomic bins (Fig. 2; Supplementary Figs S2 and S3). These enrichments decreased significantly when we examined the distribution of mirror repeats across chromosomes, indicating that the subset of H-DNA motifs are driving the observed enrichment at rDNA arrays (Fig. 2; Supplementary Figs S2 and S3).

Across the acrocentric chromosomes 13, 14, 15, 21, and 22, we report that 54.82% of all H-DNA motifs found in these chromosomes are in rDNA array loci. Specifically, in chromosomes 13 and 21, 66% and 69% of H-DNA motifs are found in the rDNA array loci, even though these regions represent a minority of the genomic space of the chromosome. In contrast, the non-acrocentric human chromosomes did not exhibit such pronounced and extended clusters of H-DNA motifs in consecutive genomic bins (Fig. 2; Supplementary Figs S2 and S3). These results indicate that H-DNA motifs are not uniformly distributed across the human genome or within individual chromosomes. Instead, they form highly localized clusters, with a particularly pronounced enrichment at rDNA arrays.

### H-DNA is most enriched at rDNA arrays

**Figure 3.**
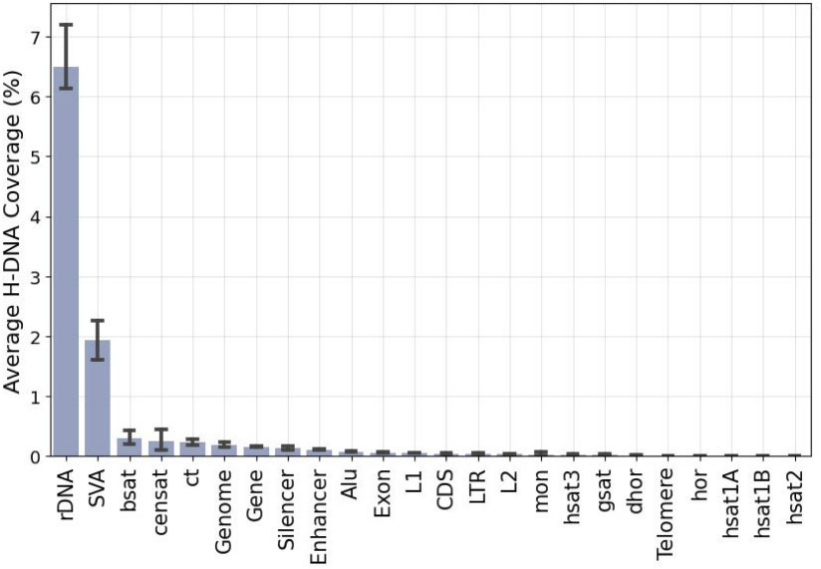
H-DNA motif coverage across human genome subcompartments and repeats. Repeats include inactive αSat HOR (hor), divergent αSat HOR (dhor), monomeric αSat (mon), classical human satellite 1A (hsat1A), classical human satellite 1B (hsat1B), classical human satellite 2 (hsat2), classical human satellite 3 (hsat3), β-satellite (bsat), γ-satellite (gsat), other centromeric satellites (censat), centromeric transition regions (ct), and telomeric regions. Error bars show the 99% confidence interval for average coverage across instances of each repeat type.

Repetitive elements comprise ∼50% of the human genome [32]. We were interested to investigate if there are specific genomic elements in which H-DNA motifs form hotspots and compare our findings to the enrichment we observed for rDNA arrays. We aimed to explore the highly repetitive elements recently elucidated by the T2T consortium. We observed that most H-DNA motif hotspots indeed coincided with rDNA array annotations (Fig. 3). Even though rDNA array loci represent 0.31% of the human genome, we find that they harbor 12.69% of the total H-DNA motifs. This represents a 40.94-fold enrichment to what would be expected by a random distribution. We compared the density of H-DNA motifs at rDNA loci relative to other genomic subcompartments. The compared elements included several types of centromeric and pericentromeric repeats, genic regions, enhancers, silencers, rDNA arrays, and telomeres. We find that the H-DNA density is orders of magnitude higher at the rDNA loci than the other elements, with a density of 67.28 bps per kB (Fig. 3). The second most enriched genomic element from those examined was β-satellite repeats which showed a 107-fold times lower genomic density than rDNA arrays.

Given that H-DNA is a highly repetitive element, we analyzed the proportion of H-DNA base pairs that overlap with STR bps. To do this, we extracted STRs across the genome for unit lengths up to 9 bps (see “Materials and methods” section). Our findings indicate that, genome-wide, 65.93% of H-DNA bps are also STR bps, while at rDNA loci, this overlap is 69.08%.

We also examined the frequency of H-DNA motifs across the different types of endogenous repeat elements. Across the endogenous repeat elements, we observe the highest density of H-DNA motifs at retrotransposons, followed by short interspersed nuclear elements (SINEs) (Supplementary Fig. 4A). We further subdivided the endogenous repeat elements in their subfamilies. We find that SINE VNTR Alu (SVA) repeats have the highest H-DNA motif density, followed by Alu repeats (Supplementary Fig. 4B). Given the established enrichment of G-quadruplexes at SVA repeats [54, 55], and the evolutionary young age of many Alu repeats, we compared the occurrence of H-DNA motifs at SVA and Alu repeats in presence or absence of G-quadruplexes. We performed genome-wide extraction of G-quadruplex motifs and examined the 7380 SVA and 1 147 458 Alu repeats found in the human genome. Since the reported SVA and Alu repeat loci were at times overlapping we merged the overlapping regions, resulting in 6609 SVA and 1 146 859 Alu nonoverlapping regions. We find that 3027 H-DNA motifs are located within 1496 SVA elements which accounts for 22, 63% of the total SVA repeats. In particular, in SVA loci, 2700 out of the identified 3027 (89.2%) H-DNA sequences also overlap with a G-quadruplex sequence. In contrast, when examining the Alu elements, we identified 15 259 H-DNA sequences in 12 221 Alu repeat loci, but only 1017 out of the 15 259 (6.7%) H-DNA motifs overlap with a G-quadruplex sequence. In addition, we examined the respective coverage of the detected H-DNA and G-quadruplex motifs within SVA and Alu elements. The average H-DNA motif base pair coverage was estimated at 1.93% and 0.07% of the total SVA and Alu sequence space, respectively (Supplementary Fig. 5A–D). Interestingly, we report a total of 28 SVA and 96 Alu elements that are covered entirely by H-DNA sequences (Supplementary Fig. 5C and D). The most H-DNA rich Alu repeats do not contain any G-quadruplex motifs, whereas in SVA repeats a significant subset contains H-DNA and G-quadruplex motifs that overlap one another. We conclude that H-DNA motifs are highly enriched at SVA repeats and tend to coincide with G-quadruplexes. These findings are particularly interesting since SVA retrotransposons and Alu repeats are evolutionary young and still active in the human genome [56, 57]. Nevertheless, the observed H-DNA densities at endogenous repeats remained significantly lower than the density at rDNA arrays (Supplementary Fig. S4B and Fig. 3). We conclude that across the genomic elements examined rDNA is the element with the highest, on average, H-DNA density.

### Intergenic loci at rDNA arrays are hotspots for H-DNA

**Figure 4.**
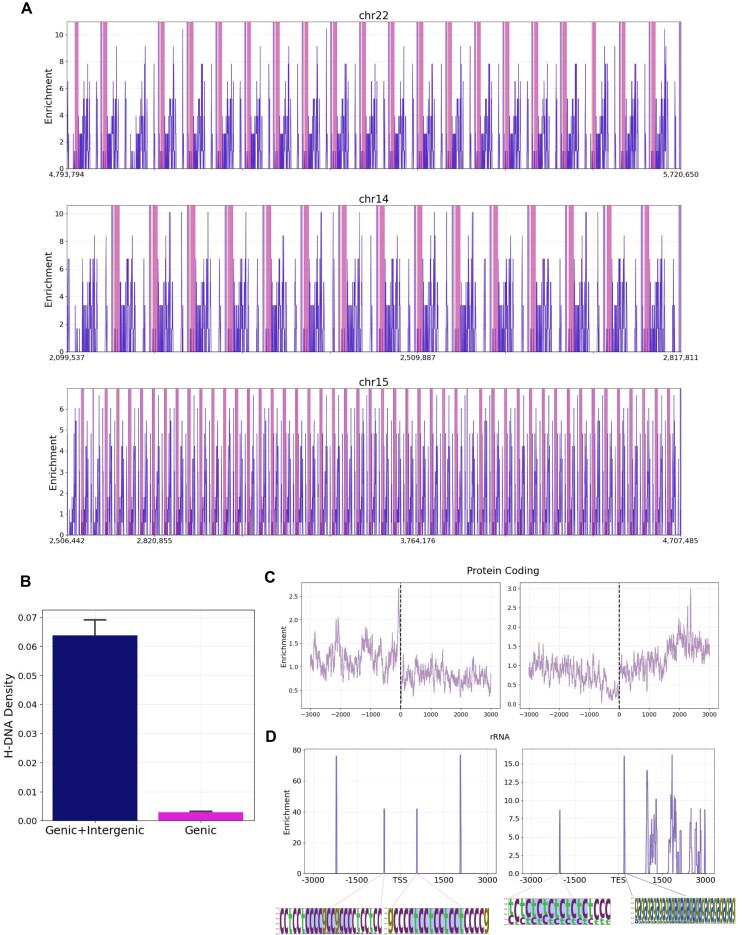
Density of H-DNA in genic, intergenic regions of rDNA arrays. (**A**) Enrichment of H-DNA motifs in rDNA arrays in chromosomes 22, 14, and 15. Marked in pink is the location of genes. (**B**) Distribution of H-DNA motifs in genic, intergenic regions of rDNA arrays versus in genic regions. (**C** and **D**) H-DNA enrichment relative to the TSS and the TES of (C) protein coding genes and (D) H-DNA motifs relative to TSS, TES, or rDNA arrays. The H-DNA sequences at individual peaks are displayed as positional weight matrices.

The rDNA encompasses the broader region of ribosomal encoding genes, which is highly repetitive and consists of three coding regions: 18S, 5.8S, 28S rRNA, and intergenic spacers. As a next step in our analysis, we wanted to examine the positioning of H-DNA motifs within the rDNA tandem array. We examined if H-DNA motifs were more likely to occur in the rDNA genic regions or in the intervening intergenic regions. To that end, we partitioned the rDNA tandem array in rRNA genic regions and intergenic regions and examined the H-DNA density in each respective compartment. The H-DNA signal predominantly originated from the intergenic rather than the rRNA coding, genic regions (Fig. 4A and B). We also examined the positioning of H-DNA motifs by partitioning the broader rDNA array in 3500 equally sized bins. The highest number of occurrences of H-DNA motifs were observed in the broader Intergenic spacer (IGS) region downstream of the 28S. Notably, upstream of the 18S genic regions H-DNA are depleted, with re-emergence in proximity to the 28S of the previous adjacent coding array (Fig. 4A and B).

Due the high repetitiveness of the rDNA array, the H-DNA motif enrichment phenomenon has a highly predictable periodicity, varying between each chromosome, which is suggestive of the putative regulatory role of H-DNA in rRNA coding genes. We find length 1 being the predominant spacer length of H-DNA motifs in rDNA array loci. Upon examining the extracted H-DNA motifs which emerged in the rDNA array, we noticed that the most frequent H-DNA motif in rDNA arrays, ctctctctgtctgtctctctc, which occurred 1044 times amongst the examined chromosomes, only appeared 24 times in other parts of the genome (Supplementary Table S2).

To further investigate the potential regulatory roles of H-DNA at rRNA coding genes and to obtain a more nuanced representation of the H-DNA motif distribution in relation to the TSS and TESs, we expanded the respective loci downstream of 28S and upstream of 18S in a window of 3000 bp. The resulting H-DNA distribution verified that most H-DNA motifs are found at 1000 bp downstream of the TES, while the genic area included only a few positions enriched in H-DNA (Fig. 4C). To investigate if this phenomenon is exclusive to rRNA, we examined the H-DNA distribution of TSS/TES in protein coding genes. The resulting distribution indicated that there is no significant enrichment of H-DNA motifs relative to TESs of protein coding genes (Fig. 4C). These findings indicate a highly biased distribution of H-DNA across the rDNA arrays, with clear preferential positioning, which suggest putative roles of H-DNA motifs in regulating rDNA expression.

### Examination of PRDM9 binding at H-DNA motifs

PRDM9 is a zinc finger protein with central roles in determining the location of recombination hotspots across different mammals, including humans [34, 58]. It also mediates programmed DNA double-strand breaks that result in genetic exchange between chromosomes. Previous work has identified the binding motifs of PRDM9B allele (hereafter referred to as PRDM9B) using chromatin immunoprecipitation with sequencing experiments [47] and shown that PRDM9B binding sites are enriched at rDNA arrays [39]. Additionally, H-DNA is known to be intrinsically mutagenic and cause double-strand breaks [21, 23].

**Figure 5.**
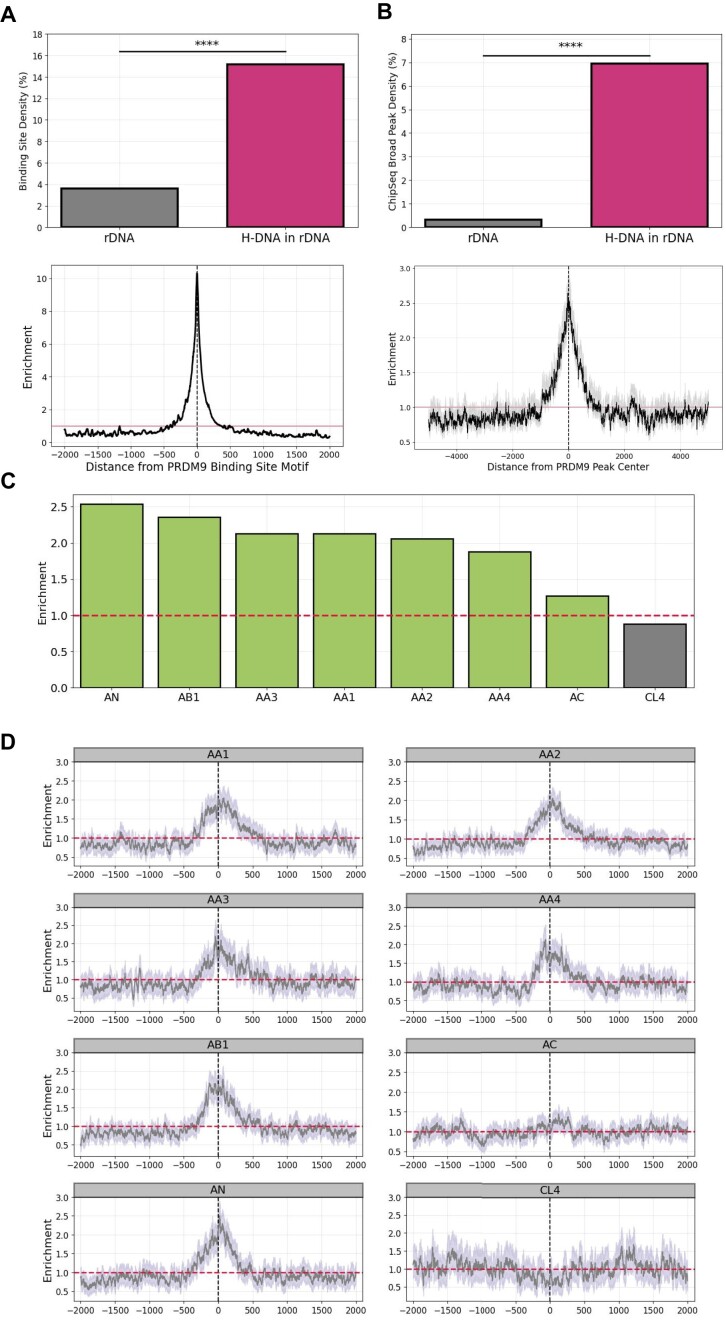
Enrichment of RDM9 motifs at H-DNA loci. (**A**) Predicted PRDM9B binding site motif absolute density in rDNA satellite regions, the percentage of H-DNA of binding site motifs that lie within rDNA. H-DNA distribution relative to predicted PRDM9B binding site motif. (**B**) PRDM9B ChIP-seq broad peak absolute density in rDNA satellite regions, the percentage of H-DNA of broad peaks that lie within rDNA. H–DNA distribution relative to ChIP-seq broad peak center. Error bars display the 1% and 99% percentiles from bootstrapping with replacement (*N* = 1000). (**C**) Maximum enrichment of H-DNA within the 2 kB interval around experimentally derived PRMD9 peaks for individuals with various alleles. Maximum enrichments that occur within the 250 bp interval around the experimentally derived PRMD9 peaks appear in green, whereas in gray appear maximum enrichments that lie outside of the 250 bp interval around the PRMD9 peak. (**D**) Positional enrichment of H-DNA motifs relative to the middle of the experimentally derived PRMD9 peaks for different combinations of alleles within a 2 kB window. We created a 95% confidence interval (light gray) around each base pair, using bootstrap *N* = 1000, with the lower end representing the 0.025 quantile, the top end representing the 0.975 quantile.

We hypothesized that PRDM9 binding sites could be enriched at H-DNA motifs because both PRDM9 and H-DNA are preferentially found at rDNA arrays and are linked to double-strand breaks, PRDM9 through its role in meiotic recombination and H-DNA due to its association with increased genomic instability. We examined the genome-wide distribution of H-DNA motifs relative to predicted PRDM9B-binding motifs and found that H-DNA motifs are over 7.16-fold enriched in predicted PRDM9B-binding sites relative to background rates (Fig. 5A). We also examined the frequency of predicted PRDM9B-binding sites across the rDNA arrays relative to the H-DNA motif loci. We report that 26.33% of predicted PRDM9B-binding sites at rDNA loci overlap at least one H-DNA motif and are significantly more likely to overlap H-DNA motifs than expected (Fisher’s exact test, *P*-value < 0.0001; Fig. 5A). To validate our findings we re-analyzed the PRDM9B ChIP-seq data from Altemose *et al.* [47]. We conducted the ChIP-seq peak calling anew for CHM13v2 both genome-wide and after adjusting the read mapping parameters for the highly repetitive rDNA loci (see “Materials and methods” section). When examining the relationship of the genome-wide experimentally determined PRDM9B peaks with H-DNA motifs, we find that 5.05% of them overlapped with at least one H-DNA sequence. Additionally, we report that 17 194 out of the 62 003 PRDM9B ChIP-seq peaks had at least one H-DNA in window of ±5 kB and showed a 2.63-fold enrichment relative to the background, suggesting that H-DNA sequences are associated with experimentally determined PRDM9B-binding sites (Fig. 5B). We also found 56 shared peaks (with 75% reciprocal overlap between replicates) located within the rDNA satellite regions, with 7.45% these bps also being H-DNA sequences, while the PRDM9B ChIP-seq peak bps themselves occupied only 0.33% of the rDNA regions (Fig. 5B), indicating strong consistency in our findings. To determine the statistical significance of the results we performed a Fisher’s exact which revealed a *P*-value of <0.005.

Next, we were interested to examine if there are differences in the enrichment of PRDM9 binding at H-DNA motifs depending on the PRDM9 allele. We analyzed ChIP-seq data derived from normal testis samples from eight individuals with different PRDM9 alleles [35]. The PRDM9 alleles examined included A, B, C, N, and L4, which can be grouped in two categories with similar characteristics: A-like alleles (including A, B, and N alleles) and C-like alleles (including C and L4 alleles) [35]. In the individuals examined, we report substantial differences in the enrichment levels of H-DNA motifs in experimentally determined PRDM9 binding of individuals with different PRDM9 alleles, ranging between 2.5-fold (AN) and 0.88-fold (CL4), with a mean enrichment of 1.90-fold (Fig. 5C and D). We also note that among the eight individuals, only for CL4 and we see a relative depletion of experimentally determined PRDM9 binding directly at the H-DNA motif sites. These findings indicate that A-like alleles (including A, B, and N alleles) PRDM9-binding sites are enriched in H-DNA motifs, whereas PRDM9 C-like alleles (including C and L4 alleles) are not. Since H-DNA can cause DNA double strand breaks, this could indicate novel biological roles of non-B DNA in mediating meiotic recombination but the lack of enrichment at PRDM9 C-like alleles indicates that more work is required to examine if the H-DNA structure formation impacts the genetic exchange between chromosomes during meiosis.

### H-DNA is highly enriched in rDNA arrays across great ape genomes

Finally, we were interested to investigate if our findings regarding the enrichment of H-DNA in rDNA arrays were also present in other primate species. To provide evidence for the enrichment of H-DNA across great apes, we utilized the T2T primate genomes [40] recently sequenced and examined the distribution of H-DNA in the genomes of six nonhuman primates including *Gorilla gorilla* (gorilla), *Pan paniscus* (bonobo), *Pan troglodytes* (chimpanzee), *Pongo abelii* (Sumatran orangutan), *Pongo pygmaeus* (Bornean orangutan), and *Symphalangus syndactylus* (Siamang gibbon).

**Figure 6.**
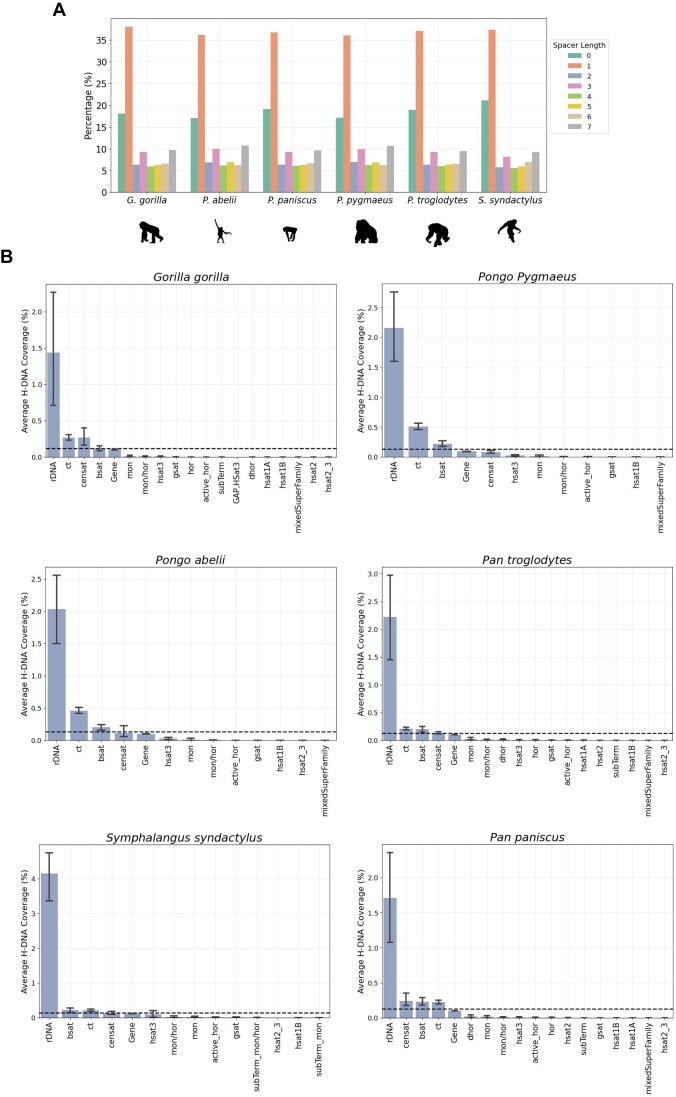
Distribution of H-DNA in T2T primate genomes. (**A**) Percentage of H-DNA motifs as a function of spacer length. (**B**) Repeats include inactive αSat HOR (hor), divergent αSat HOR (dhor), monomeric αSat (mon), classical human satellite 1A (hsat1A), classical human satellite 1B (hsat1B), classical human satellite 2 (hsat2), classical human satellite 3 (hsat3), β-satellite (bsat), γ-satellite (gsat), other centromeric satellites (censat), and centromeric transition regions (ct). The horizontal line in (B) represents the genomic density of H-DNA.

In these nonhuman great ape genomes, the number of mirror repeats per diploid genome varies from 2 287 356 to 2 293 537 in chimpanzee and gorilla species respectively, with a median count of 2 329 114.0. For H-DNA motifs, we find that their occurrences per genome ranges between 389 259 and 418 878 in Bonobo and Siamang gibbon species respectively, with a median number of H-DNA motifs observed being 397 205.5. Additionally, we find that similarly to humans, for both mirror repeats and H-DNA, repeats with spacer lengths of 0 and 1 bps are the most frequent (Fig. 6A and Supplementary Fig. S6). For both mirror repeats and H-DNA the vast majority of repeats are those with shorter arm lengths and the numbers decline precipitously with increased arm length (Supplementary Fig. S6). However, we observe that a subset of H-DNA motifs has extremely large lengths, in several cases exceeding 1000 bps (Supplementary Table S3). The largest H-DNA motifs are frequently overlapping microsatellite repeats, which is expected considering the formation of H-DNA secondary structures at such loci, with polypurine/polypyrimidine mirror repeat symmetry [59].

Next, we examined the distribution of H-DNA across different types of genomic compartments and repetitive elements in the great ape genomes. We find consistent results with the analysis performed for the human genome, with H-DNA motifs having the highest density at rDNA loci, across great apes, relative to centromeric and pericentromeric repeats, genic regions and telomeres (Fig. 6B). However, it should be noted that the genomic annotations of low complexity regions in these genomes may not be as accurately resolved as those in the human genome. Future research is necessary to precisely quantify the enrichment levels of these regions in comparison to other genomic areas. We conclude that rDNA is highly enriched in H-DNA across Great Apes.

## Discussion

Here, we analyzed the T2T reference human genome and found that H-DNA motifs are not homogeneously distributed between and within human chromosomes. We observe that acrocentric chromosomes show some of the highest H-DNA motif enrichments. We provide evidence which indicates that rDNA array loci, at acrocentric chromosomes, are H-DNA motif hotspots, with particularly high density of H-DNA motifs in both human and other great ape primate genomes. When comparing the H-DNA motif density in rDNA arrays to that found in other genomic elements, we observe that rDNA arrays have the highest H-DNA motif density. Interestingly, H-DNA is localized in the intergenic spacer regions between the regions expressing rRNA. We also observe that H-DNA motifs are ∼2.5-fold enriched at binding sites of PRDM9A-like alleles. It is however unclear whether this feature might play a role in meiotic recombination, given that this enrichment is not observed for PRDM9C-like alleles and further work is required to characterize the potential interplay between specific PRDM9 alleles and H-DNA.

As the ribosome is pivotal in protein assembly, RNAPI accounts for the synthesis of ∼60% of cellular RNA [60]. Our discovery of H-DNA motif abundance at rDNA arrays leads us to hypothesize that H-DNA formation has regulatory effects on rRNA expression. Intermolecular triplexes have been shown to form at rDNA loci [30]. Nevertheless, future work is needed to examine the effects of the formation of H-DNA structures at rDNA loci and decipher their regulatory roles. It will also be important to determine whether H-DNA acts as a regulator of RNAPI and if the positioning of H-DNA motifs impact its activity.

H-DNA is a known mutational hotspot and is associated with genomic instability. Previous studies have shown associations between H-DNA formation and increased mutation rate, as well as links with human diseases [2, 21, 23, 61–63]. Given the essential role of rDNA in maintaining cellular homeostasis, future research should investigate whether mutations acquired during cancer development or associated with other human diseases at H-DNA sites within rDNA arrays interfere with ribosomal RNA expression.

Furthermore, the enrichment of H-DNA motifs at binding sites of PRDM9 A-like alleles (including A, B, and N alleles) but not C-like alleles (including C and L4 alleles) may indicate an involvement of intramolecular triple-stranded DNA structures in meiotic recombination for specific PRDM9 alleles. Previous work has shown that H-DNA causes double-strand breaks *invivo* [21, 23]. Physiological roles of H-DNA formation for the generation of double-strand breaks during meiotic recombination, via specific alleles of PRDM9, is an attractive hypothesis and more work is required to investigate if such mechanisms are modulating meiotic recombination.

Finally, as more T2T genomes are assembled from species of different taxonomic groups, future research will have greater opportunities to uncover the role of H-DNA in influencing eukaryotic evolution and adaptation, particularly as it pertains in the regulation of rDNA expression.

## Supplementary Material

lqaf012_Supplemental_Files

## Data Availability

The GitHub code and all the related material is provided at https://github.com/Georgakopoulos-Soares-lab/hdna_rdna_homo_sapiens and in Zenodo https://zenodo.org/records/14829698.
