# Supplementary material for: Ribosomal DNA arrays are the most H-DNA rich element in the human genome
Source: NAR Genom Bioinform. 2025 Mar 4;7(1):lqaf012. doi: 10.1093/nargab/lqaf012 (PMC11879447; doi:10.1093/nargab/lqaf012)
Supplement: lqaf012_Supplemental_Files [file lqaf012_supplemental_files.zip › Mirror Repeats Manuscript_revision_2_supplementary.pdf]

Supplementary Material

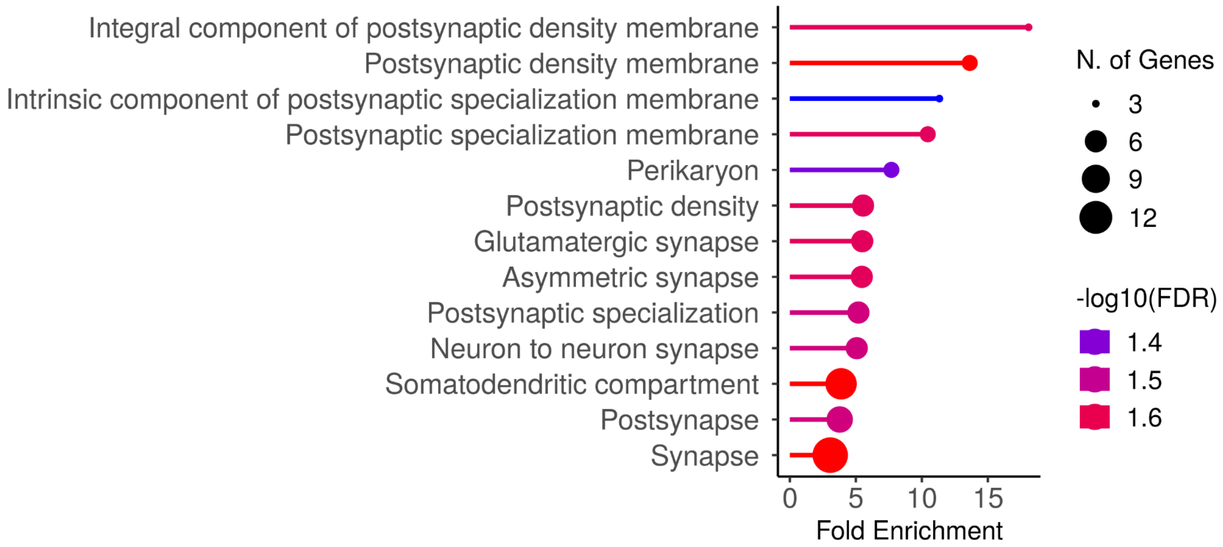

Supplementary Figure 1: GO Cellular Component pathway analysis for genes overlapping H-DNA sequences with arm lengths of at least 60bps.

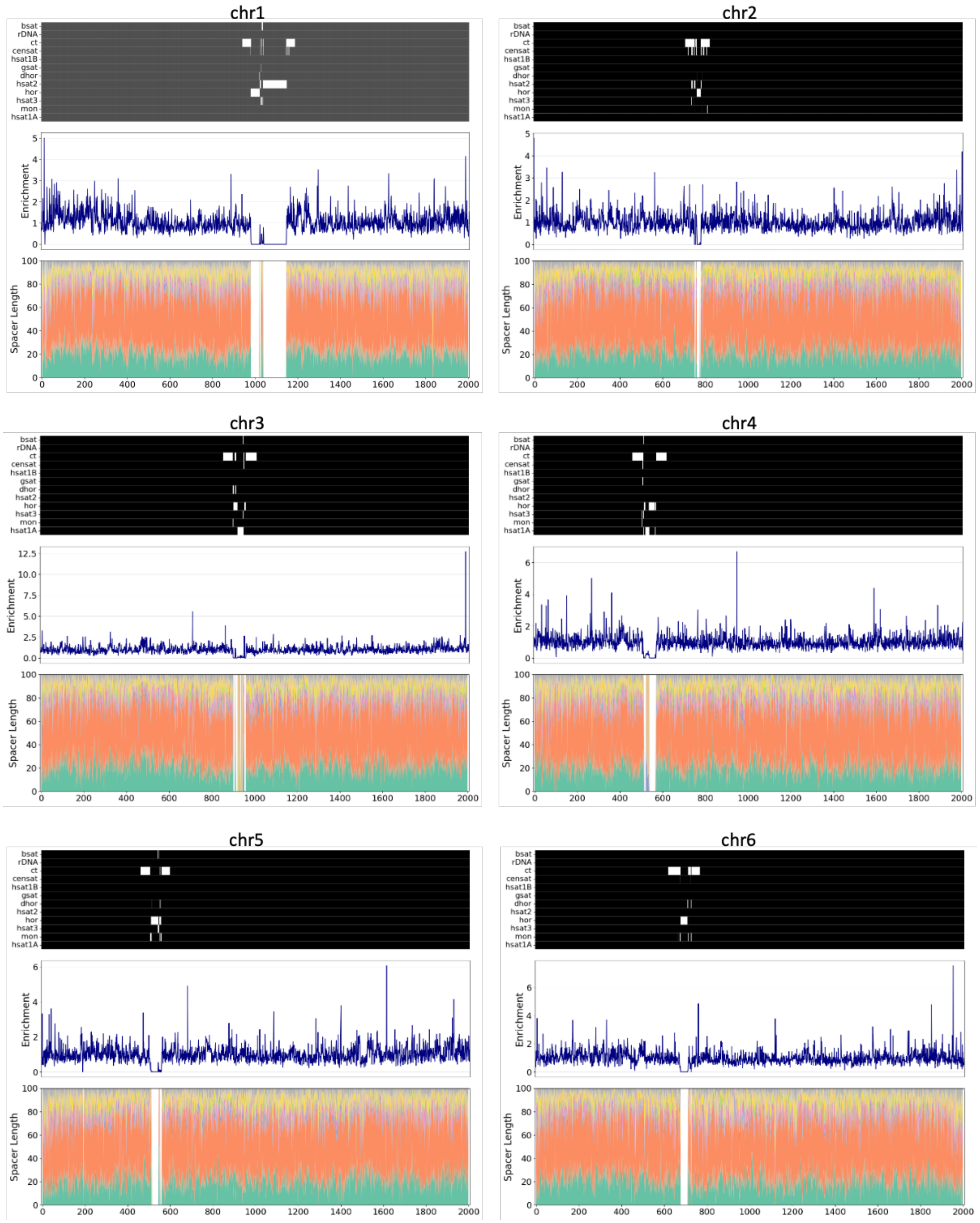
chr1
chr2
chr3
chr4
chr5
chr6
bsat
rDNA
ct
censat
hsat1B
gsat
dhor
hsat2
hor
hsat3
mon
hsat1A
Enrichment
Spacer Length

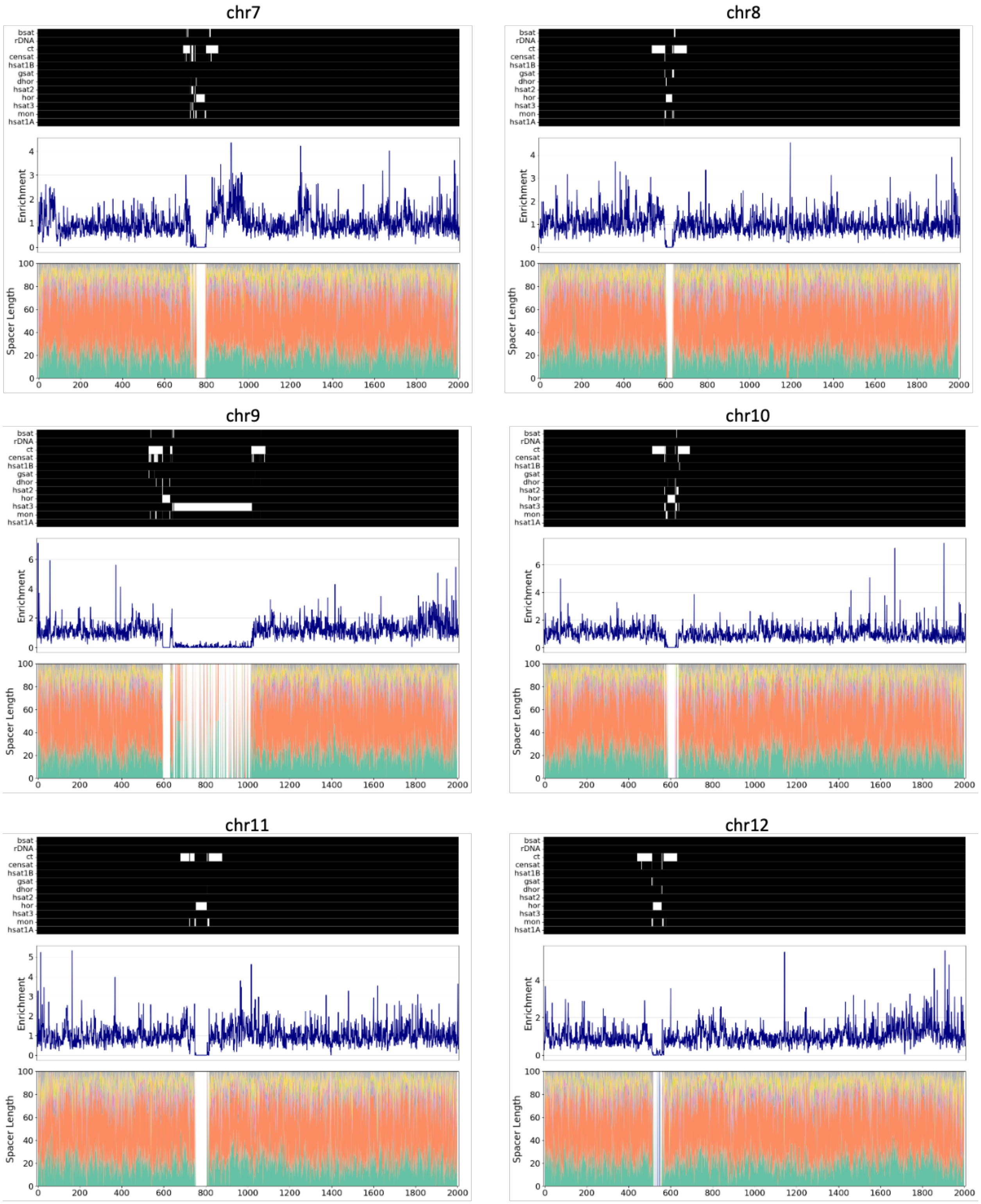

chr7
chr8
chr9
chr10
chr11
chr12
bsat
rDNA
ct
censat
hsat1B
gsat
dhor
hsat2
hor
hsat3
mon
hsat1A
Enrichment
Spacer Length
0
200
400
600
800
1000
1200
1400
1600
1800
2000

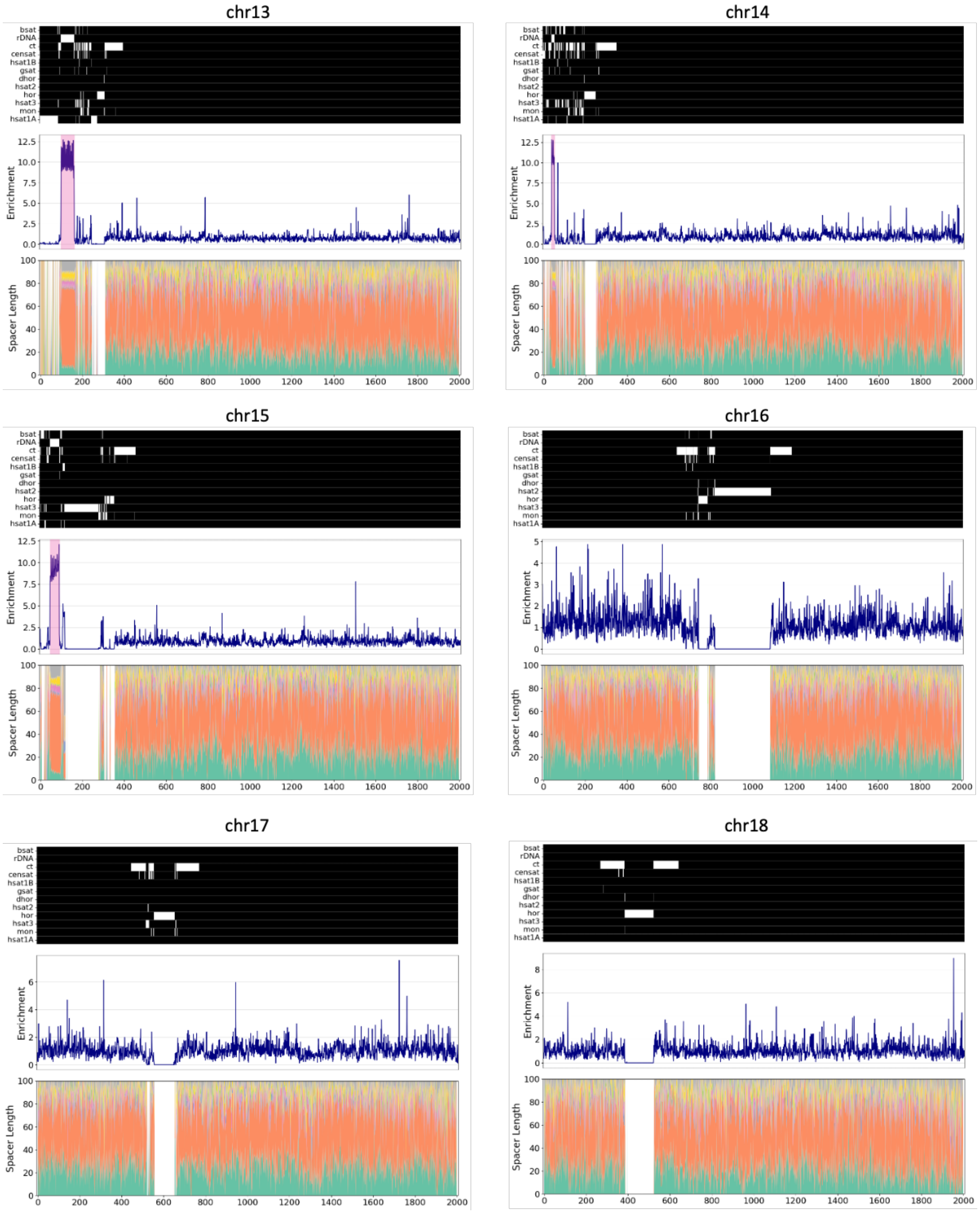
chr13
chr14
chr15
chr16
chr17
chr18
bsat
rDNA
ct
censat
hsat1B
gsat
dhor
hsat2
hor
hsat3
mon
hsat1A
Enrichment
Spacer Length
0
200
400
600
800
1000
1200
1400
1600
1800
2000

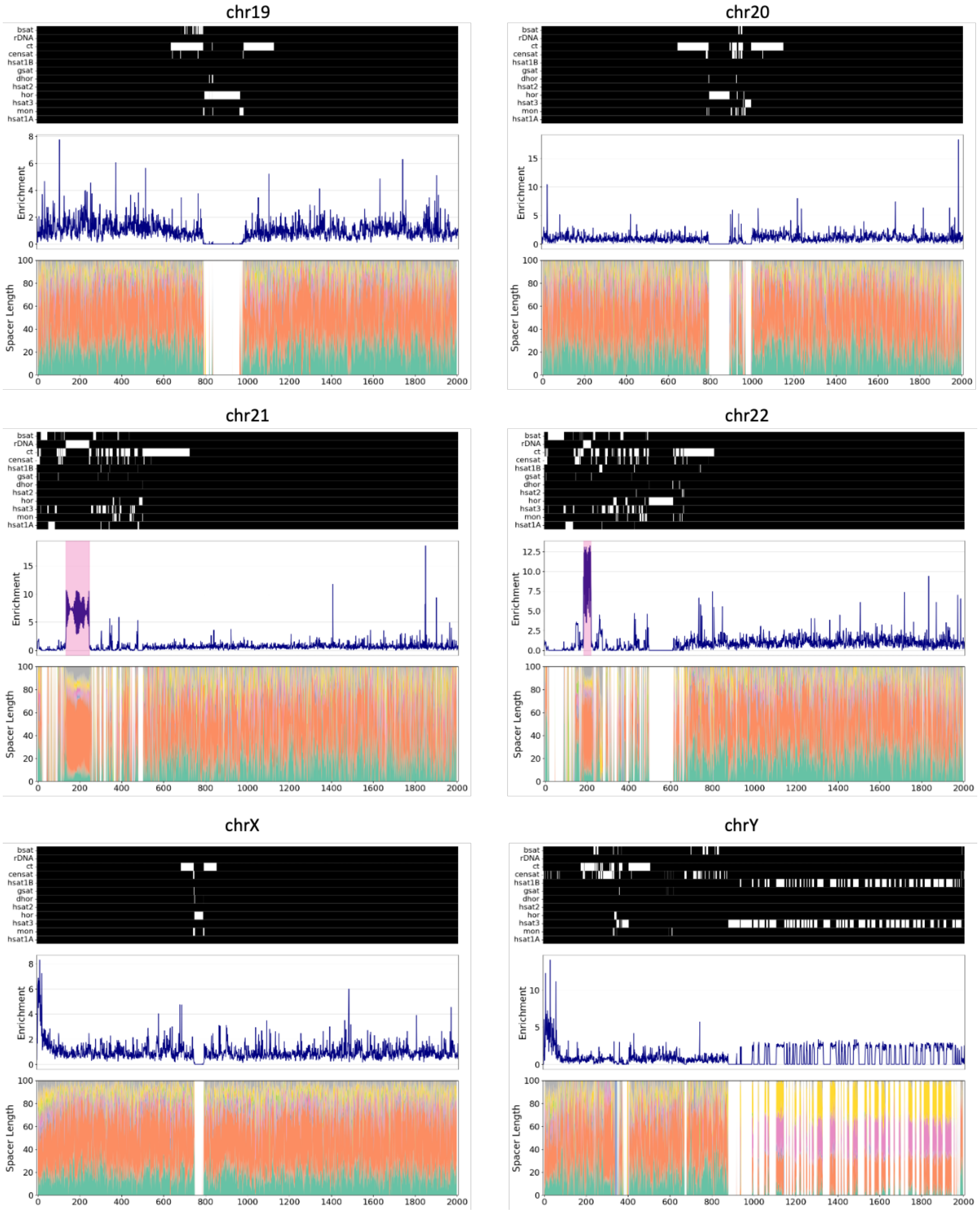

Supplementary Figure 2: Mirror repeat profile in different human chromosomes. Schematics show the distribution of mirror repeats across different human chromosomes. The heatmap shows the different types of pericentromeric and centromeric repeats as well as rDNA arrays, with white color representing presence in that genomic region. Line plots show the mirror

repeat enrichment at each genomic bin for a chromosome. Colored in pink are highlighted the rDNA array loci. Repeats include inactive αSat HOR (hor), divergent αSat HOR (dhor), monomeric αSat (mon), classical human satellite 1A (hsat1A), classical human satellite 1B (hsat1B), classical human satellite 2 (hsat2), classical human satellite 3 (hsat3), beta satellite (bsat), gamma satellite (gsat), other centromeric satellites (censat) and centromeric transition regions (ct).

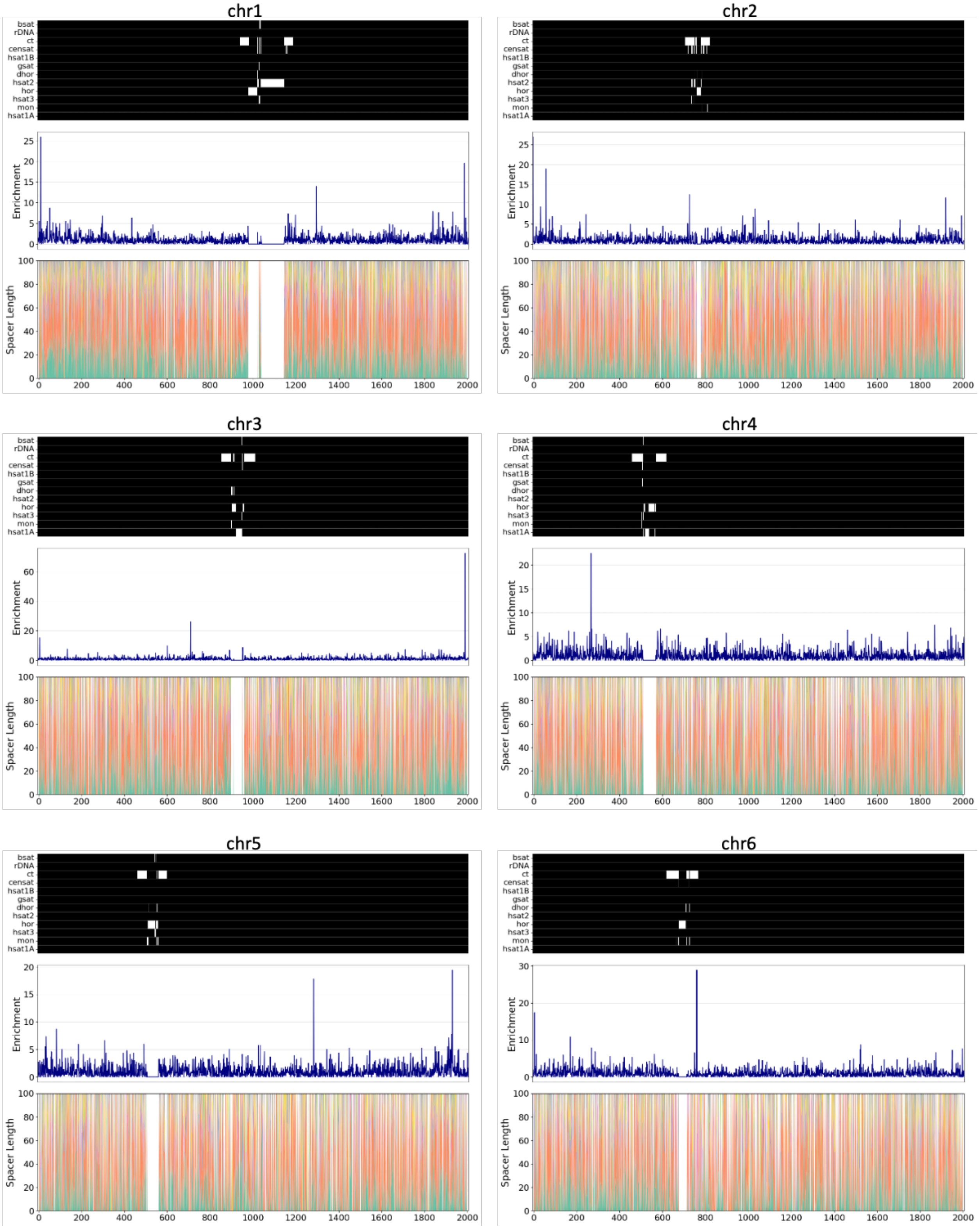
chr1
chr2
chr3
chr4
chr5
chr6
bsat
rDNA
ct
censat
hsat1B
gsat
dhor
hsat2
hor
hsat3
mon
hsat1A
Enrichment
Spacer Length

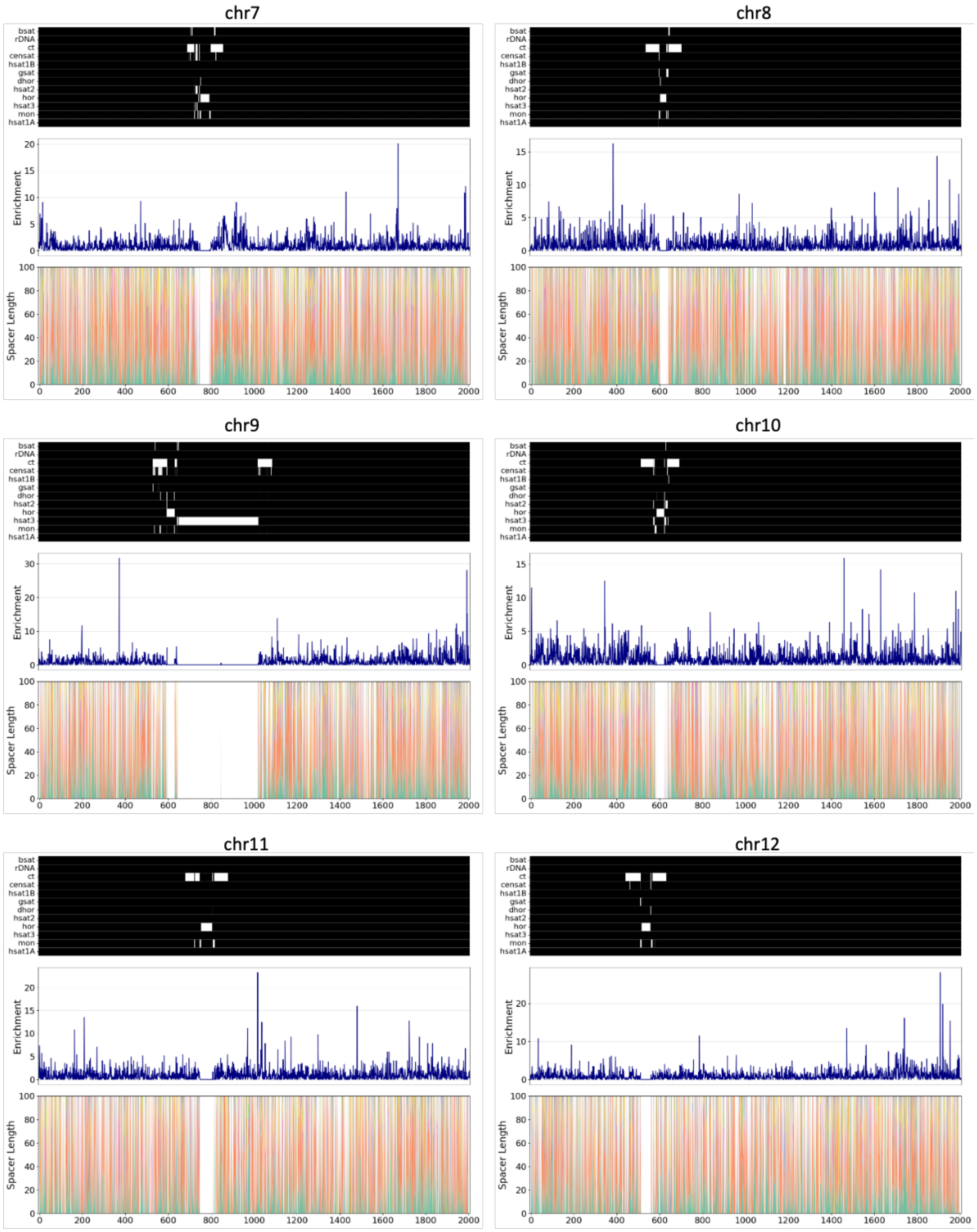
chr7
chr8
chr9
chr10
chr11
chr12
bsat
rDNA
ct
censat
hsat1B
gsat
dhor
hsat2
hor
hsat3
mon
hsat1A
Enrichment
Spacer Length

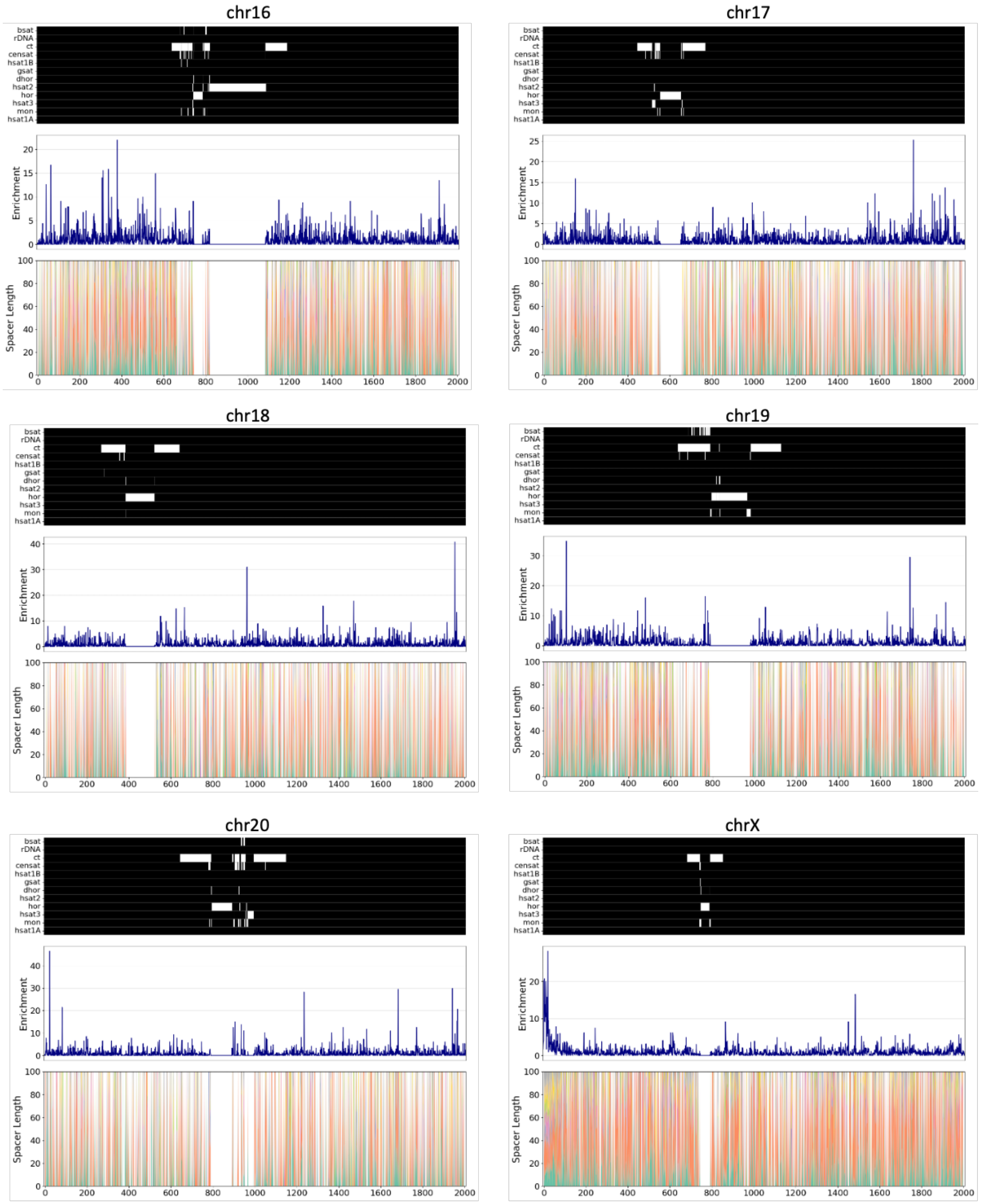

Supplementary Figure 3: H-DNA profile in different human chromosomes. Schematics show the distribution of H-DNA motifs across different human chromosomes. The heatmap shows the different types of pericentromeric and centromeric repeats as well as rDNA arrays, with white color representing presence in that genomic region. Line plots show the H-DNA motif enrichment

at each genomic bin for a chromosome. Colored in pink are highlighted the rDNA array loci. Repeats include inactive αSat HOR (hor), divergent αSat HOR (dhor), monomeric αSat (mon), classical human satellite 1A (hsat1A), classical human satellite 1B (hsat1B), classical human satellite 2 (hsat2), classical human satellite 3 (hsat3), beta satellite (bsat), gamma satellite (gsat), other centromeric satellites (censat) and centromeric transition regions (ct).

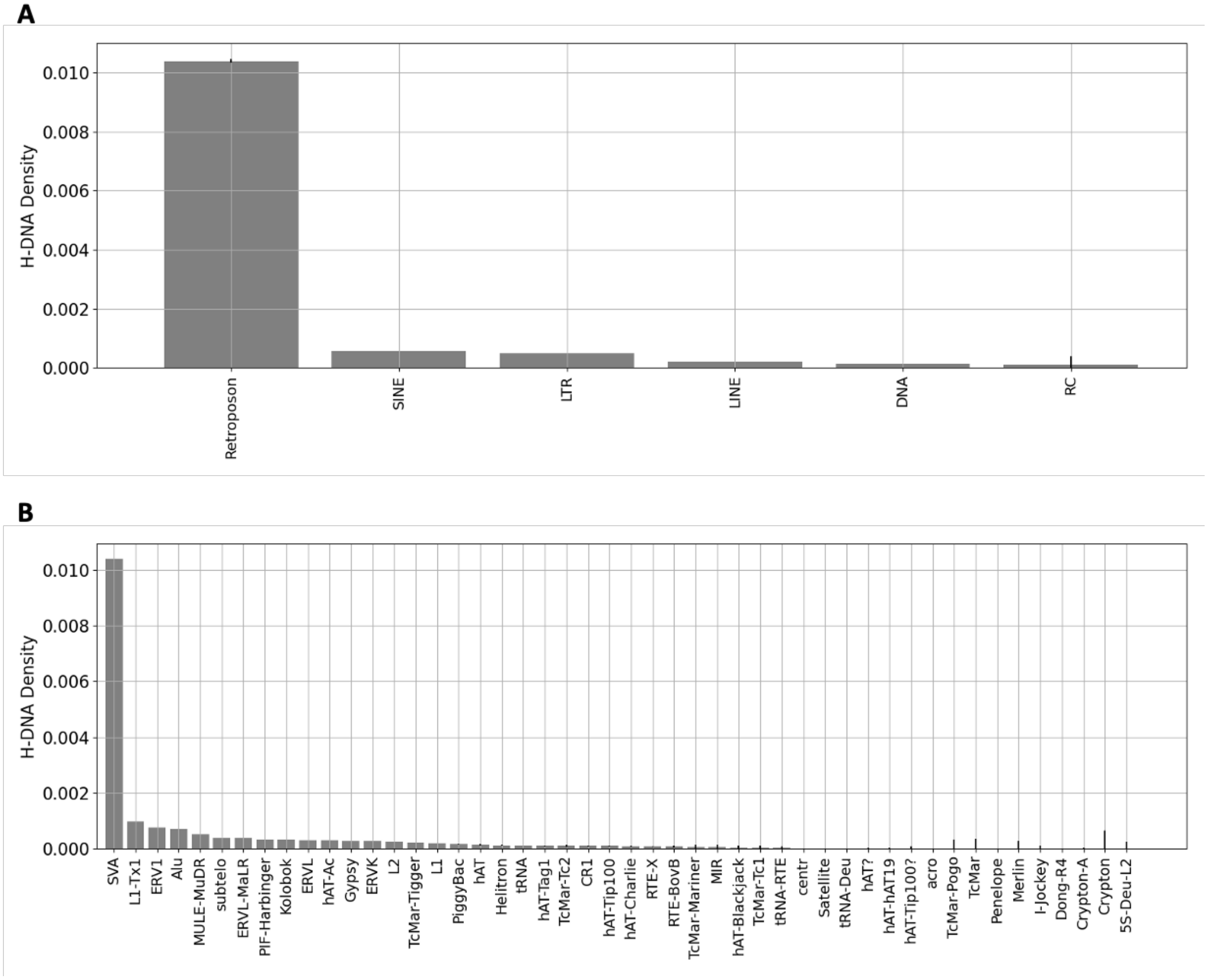

Supplementary Figure 4: H-DNA Density across transposable elements in the CHM13 reference human genome. A. Distribution of G4s in transposable elements and satellite repeats. **B.** Distribution of G4s in transposable elements sub-categories. Error bars represent standard deviation from bootstrapping with replacement (N=1,000).

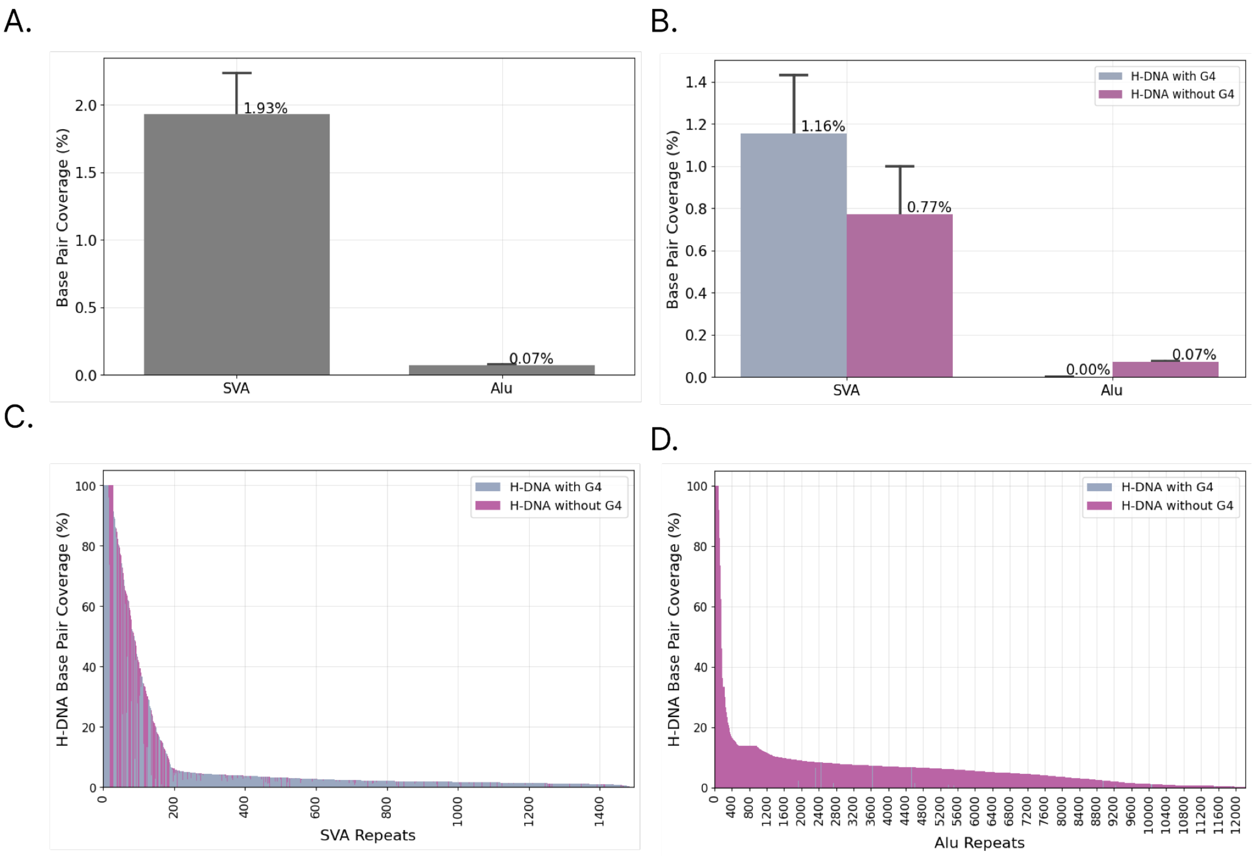

Supplementary Figure 5: H-DNA and G-quadruplex motifs at Alu and SVA repeats. A. Average H-DNA base pair coverage (%) of SVA and Alu transposable elements. **B.** Average H-DNA base pair coverage (%) with and without G4 sequences of SVA and Alu elements. **C.** H-DNA base pair coverage (%) with and without G4 of SVA elements at least one H-DNA in decreasing order. **D.** H-DNA base pair coverage (%) of Alu elements with at least one H-DNA partitioned on H-DNA with and without G4 sequences in decreasing order. The error bars represent 99% confidence intervals for the mean.

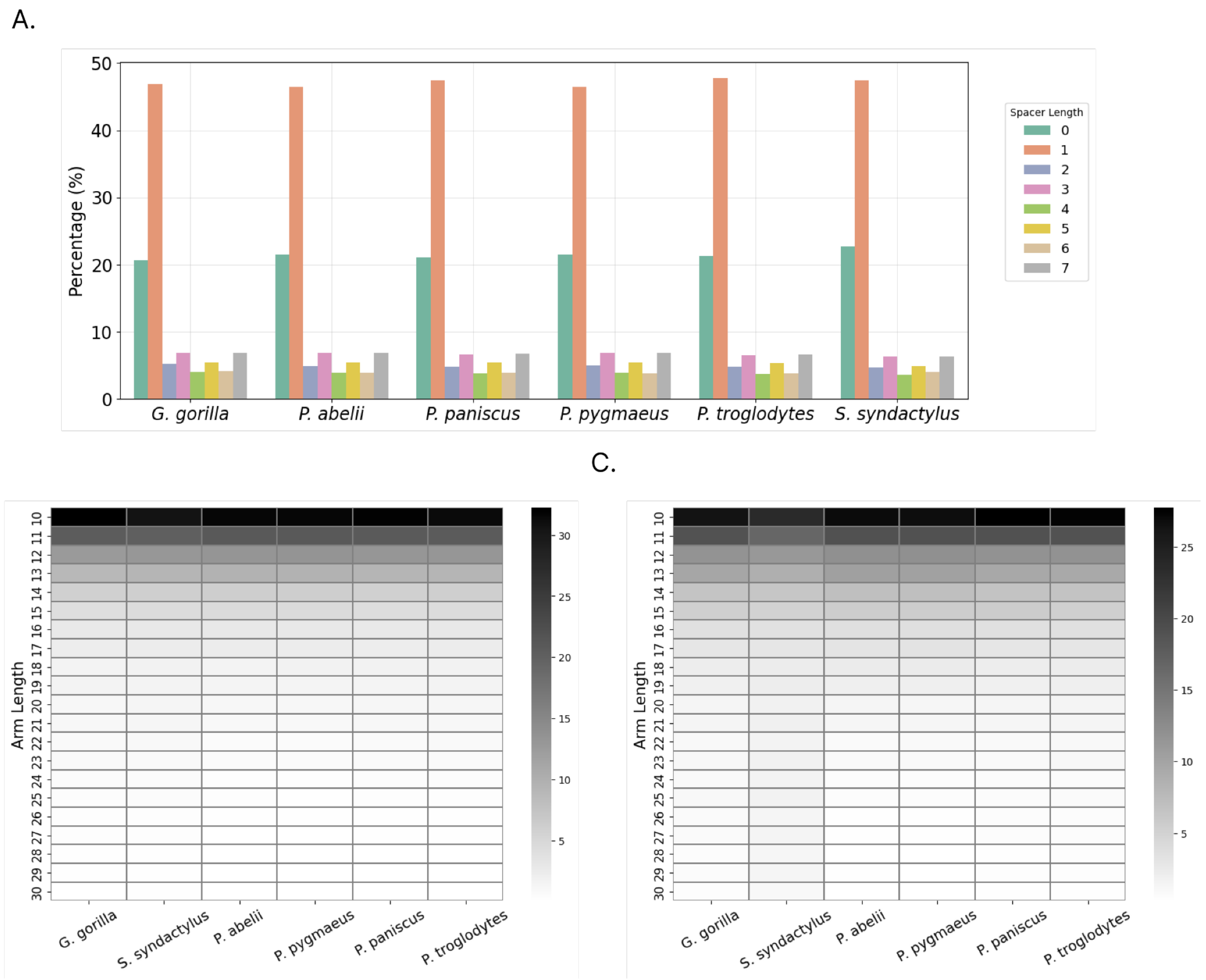

Supplementary Figure 6: Sequence analysis of mirror repeats and H-DNA motifs. A. Percentage of mirror repeats as a function of spacer length. **B.** Percentage of mirror repeats as a function of arm length. **C.** Percentage of H-DNA motifs as a function of arm length.

Supplementary Table 1: Largest H-DNA motifs and the genes they overlap.

Chromosome	H-DNA Start	H-DNA End	Arm Length	Gene Name	Biotype	Overlap
chr1	39532901	39533697	398	*NT5C1A*	Protein Coding	796
chr18	666958	667375	208	*LINC01925*	lncRNA	417
chr20	52435793	52436194	200	*RIPOR3*	Protein Coding	401
chr18	49776752	49777085	166	*LIPG*	Protein Coding	333
chr18	64594572	64594903	165	*LINC01924*	lncRNA	331
chr1	216765929	216766253	162	*GPATCH2*	Protein Coding	324
chr5	170487803	170488126	161	*INSYN2B*	Protein Coding	323
chr5	170487803	170488126	161	*DOCK2*	Protein Coding	323
chr4	153982331	153982646	157	*LRBA*	Protein Coding	315
chr17	81566759	81567056	148	*RPTOR*	Protein Coding	297
chr12	95884963	95885246	141	*CCDC38*	Protein Coding	283
chr8	97376118	97376394	134	*MTERF3*	Protein Coding	273
chrX	106347502	106347765	131	*LOC101928335*	lncRNA	263
chrX	106347502	106347765	131	*MID2*	Protein Coding	263

Supplementary Table 2: H-DNA motifs with highest number of counts in human rDNA array and the intervening intergenic regions.

H-DNA Motif	Total Occurrences	Spacer Length
ctctctctgtctgtctctctc	1,044	1
agagagagagagagagagaga	853	1
agagagacagagacagagaga	695	1
ctctctctctctctctctctc	671	1
agaaagagacagacagagaaaga	667	1
agagagagagagagagagagagaga	559	1
acagagagagagagagagagaca	511	1
agagagagagagagagagagagagaga	502	1
ctctctctctctctctctctctctc	500	1
agagagagagagagagagagaga	450	1
cttttctttcattttctctttcttttc	440	7
aagaaagaaacaaaagaaagaaagaa	421	6
ctctctctcactcactctctctc	420	1
agagagagaaacagacagaaagagagaga	419	7

aaagaaagaaagaaagaaagaaagaaagaaagaaagaaagaaagaaagaaa	337	1
agaaagaaagaaagaaagaaagaaagaaagaaagaaagaaagaaagaaaga	337	1
agagagagagagagagagagagagagagagagagagaga	304	1
agagagagagagagagagagagagagagaga	300	1
agacagagagaaagagagagacaga	296	3
agagaaagagacagacagacagagaaagaga	296	1
agagagagggacagggagagaga	296	1
ctctctctttctgtctgtttctctctc	296	7

Supplementary Table 3: Longest H-DNA motifs across non-human great ape genomes.

Species	Spacer Length (bp)	Arm Length (bp)
Gorilla gorilla	0	*714*
Gorilla gorilla	1	*712*
Gorilla gorilla	1	*500*
Pan paniscus	1	*481*
Pan paniscus	0	*451*
Pan paniscus	1	*442*
Pan troglodytes	1	*205*
Pongo abelii	1	*1198*
Pongo abelii	1	*1029*
Pongo pygmaeus	7	*759*
Pongo pygmaeus	1	*525*
Symphalangus syndactylus	1	*945*
Symphalangus syndactylus	1	*404*

Supplementary Data File: Genes in which the longest H-DNA motifs are found in.
